# Multicenter Collaborative Study of the Interaction of Antifungal Combinations against *Candida* Spp. by Loewe Additivity and Bliss Independence-Based Response Surface Analysis

**DOI:** 10.3390/jof8090967

**Published:** 2022-09-16

**Authors:** Joseph Meletiadis, David R. Andes, Shawn R. Lockhart, Mahmoud A. Ghannoum, Cindy C. Knapp, Luis Ostrosky-Zeichner, Michael A. Pfaller, Vishnu Chaturvedi, Thomas J. Walsh

**Affiliations:** 1 Clinical Microbiology Laboratory, Attikon University Hospital, National and Kapodistrian University of Athens, 12462 Athens, Greece; 2 Department of Medicine, University of Wisconsin-Madison, Madison, WI 53726, USA; 3 Mycotic Diseases Branch, Centers for Diseases C, Atlanta, GA 30333, USA; 4 Center for Medical Mycology, Case Western Reserve University, Cleveland, OH 44106, USA; 5 Trek Diagnostics System, Cleveland, OH 44131, USA; 6 Division of Infectious Diseases, University of Texas Health Science Center, Houston, TX 77030, USA; 7 Medical Microbiology Division, Department of Pathology, The University of Iowa College of Medicine, Iowa City, IA 52242, USA; 8 Westchester Medical Center, New York Medical College, Valhalla, NY 10595, USA; 9 Transplantation-Oncology Infectious Diseases, Weill Cornell Medicine of Cornell University, New York, NY 10065, USA; 10 Center for Innovative Therapeutics and Diagnostics, Richmond, VA 23223, USA

**Keywords:** pharmacodynamic interactions, Loewe additivity, Bliss independence, synergy, antagonism, *Candida*, antifungal drugs

## Abstract

Combination antifungal therapy is widely used but not well understood. We analyzed the spectrophotometric readings from a multicenter study conducted by the New York State Department of Health to further characterize the in vitro interactions of the major classes of antifungal agents against *Candida* spp. Loewe additivity-based fractional inhibitory concentration index (FICi) analysis and Bliss independence-based response surface (BIRS) analysis were used to analyze two-drug inter- and intraclass combinations of triazoles (AZO) (voriconazole, posaconazole), echinocandins (ECH) (caspofungin, micafungin, anidulafungin), and a polyene (amphotericin B) against *Candida albicans*, *C. parapsilosis*, and *C. glabrata*. Although mean FIC indices did not differ statistically significantly from the additivity range of 0.5–4, indicating no significant pharmacodynamic interactions for all of the strain–combinations tested, BIRS analysis showed that significant pharmacodynamic interactions with the sum of percentages of interactions determined with this analysis were strongly associated with the FIC indices (Χ^2^ 646, *p* < 0.0001). Using a narrower additivity range of 1–2 FIC index analysis, statistically significant pharmacodynamic interactions were also found with FICi and were in agreement with those found with BIRS analysis. All ECH+AB combinations were found to be synergistic against all *Candida* strains except *C. glabrata*. For the AZO+AB combinations, synergy was found mostly with the POS+AB combination. All AZO+ECH combinations except POS+CAS were synergistic against all *Candida* strains although with variable magnitude; significant antagonism was found for the POS+MIF combination against *C. albicans*. The AZO+AZO combination was additive for all strains except for a *C. parapsilosis* strain for which antagonism was also observed. The ECH+ECH combinations were synergistic for all *Candida* strains except *C. glabrata* for which they were additive; no antagonism was found.

## 1. Introduction

Combination therapy is often considered as an alternative therapeutic approach to difficult-to-treat *Candida* infections with the hope of increasing the efficacy of antifungal therapy [1]. The availability of several systemic antifungal drugs with a distinct mode of action increased the interest in combination antifungal therapy [2]. The polyene amphotericin B acts on fungal membranes, while azoles like posaconazole and voriconazole inhibit ergosterol biosynthesis and the echinocandins caspofungin, micafungin, and anidulafungin inhibit cell wall synthesis. The distinct mode of action of each class of antifungal drugs increases the possibility of efficacious combination therapy but cannot always predict the nature and magnitude of pharmacodynamic interactions [3]. Therefore, in vitro testing may help understand the pharmacodynamic interactions of antifungal drugs against *Candida* isolates and distinguish between synergistic and antagonistic combinations.

Checkerboard titration using 96-well plates is often used to study in vitro antifungal combinations. Increasing concentrations of the drugs are combined in two-fold series of dilutions, and fungal growth is measured after 24–48 h of incubation. Several models have been developed in order to analyze checkerboard data and detect synergistic/antagonistic interactions [4,5]. The fractional inhibitory concentration index is based on the Loewe additivity theory and requires a two-fold reduction/increase of the MIC of both drugs when combined in order to detect significant pharmacodynamic interactions [6]. Although this index is often used to analyze antifungal combinations, most interactions are found to be additive since this index can detect only strong interactions. Optimization studies of this index have been published suggesting a narrower additivity range based on the analysis of several replicates [7]. Another model used to analyze antifungal drug combinations is based on the Bliss independence theory where pharmacodynamic interactions are described by the difference between the fungal growth observed and that expected if the drugs were acting independently. The latter model has been previously used to analyze combinations with drugs belonging to different antifungal classes, and in vitro–in vivo correlations have been found [8,9].

Although the two models have been compared in single-center studies, their usefulness have never been evaluated in multicenter studies [10,11,12]. Therefore, we re-analyzed the data obtained by a multicenter study of 15 antifungal combinations tested in duplicate against five Candida strains by six different centers [13,14]. The antifungal agents belonged to different classes with a distinct mode of antifungal actions. Polyenes, azoles, and echinocandins and all inter- and intraclass drug combinations were tested.

## 2. Materials and Methods

**Test organisms.** The test organisms included two *Candida albicans* (#91 and #92), one *C. glabrata* (#93), and two *C. parapsilosis* (#94 and ATCC 22019). Quality control was ensured by testing the CLSI-recommended quality control strain *C. parapsilosis* ATCC 22019. Before the tests were performed, each isolate was passed at least twice on Sabouraud dextrose agar (Hardy Diagnostics, Santa Maria, CA, USA) to ensure its purity and viability. Stock inoculum suspensions of the *Candida* spp. were obtained from 24 h cultures on Sabouraud dextrose agar at 35 °C. The turbidity of each yeast suspension was adjusted by Trek’s nephelometer following the M27-A3 guidelines [15]. On the day of the test, a working yeast suspension of approximately 1.5 × 10^3^ cells/mL was prepared in YeastOne inoculum broth (Trek).

**Antifungal agents and microdilution panels.** Frozen reference broth microdilution trays containing serial two-fold dilutions of amphotericin B (0.06 to 4 µg/mL), anidulafungin (0.03 to 2 µg/mL), caspofungin (0.06 to 4 µg/mL), micafungin (0.06 to 4 µg/mL), posaconazole (0.02 to 1 mg/L), and voriconazole (0.02 to 1 mg/L) alone and in all 15 possible two-drug combinations in an 8 × 8 checkerboard format were provided by Trek Diagnostic Systems together with the RPMI1640 medium. The trays were shipped frozen and stored at −70 °C until testing was performed. 

**Combination testing.** The frozen trays were thawed and inoculated with the working yeast suspension by the use of an appropriate multichannel pipetting device by dispensing 100 µL into each well to give a final volume of 200 μL/well. The trays were incubated at 35 °C for 48 h in a non-CO_2_ incubator. The minimal inhibitory concentrations (MICs) were determined visually as the lowest drug concentration corresponding to complete (for AMB) or prominent (for all the other drugs) growth inhibition. The absorbance of each well of the microdilution trays was then measured spectrophotometrically at 550 nm. The percentages of fungal growth in each well were calculated after subtracting background absorbance (absorbance of the well without fungi) from the absorbance of the wells and dividing by the absorbance of the drug-free control. Each of the 15 different two-drug combinations were tested in duplicate on different days in each of the six different collaborating centers against each isolate.

**Analysis.** In order to assess the nature of the in vitro interactions of each combination against each *Candida* strain, the data obtained from each center with the combination microplates were analyzed using the fractional inhibitory concentrations index which is based on the Loewe additivity no-interaction theory and the response surface model which is based on the Bliss independence no-interaction theory [4,5,6]. The Loewe additivity theory is based on the hypothesis that a drug cannot interact with itself and therefore self-drug combinations are by definition additive. In LA-based models, pharmacodynamic interactions are assessed based on the concentrations of the drugs, alone or in combination that produce the same effect. Bliss independence theory is based on the hypothesis that if two drugs do not interact and act independently, the effect can be derived by the probability law of independent events. In BI-based models, pharmacodynamic interactions are assessed comparing the combined Bliss independence effect which is calculated based on the effect of the individual drugs with the experiment.

(i) **Fractional inhibitory concentration (FIC) index analysis.** The FIC index model is expressed as ∑FIC = FIC_A_ + FIC_B_ = C_A_^comb^/MIC_A_^alone^ + C_B_^comb^/MIC_B_^alone^, where MIC_A_^alone^ and MIC_B_^alone^ are the MICs of the drugs A and B when acting alone and C_A_^comb^ and C_B_^comb^ are concentrations of the drugs A and B at the iso-effective combinations, respectively. In order to capture both synergistic and antagonistic interactions among all ∑FICs calculated for each checkerboard data set, the ∑FIC_min_ and the ∑FIC_max_ were determined as the lowest and highest ∑FIC, respectively. The MIC endpoints were defined as the lowest drug concentration showing <5% of growth compared to that of the growth control. Off-scale MICs were converted to the next-highest or -lowest doubling concentration. Finally, the median and the range of FIC indices among the replicates and centers were determined. In order to analyze statistically, the FICs were transformed to log_2_ values, and the 95% confidence interval of the FIC of all replicates and centers was calculated for each drug combination and strain based on the *t* distribution. When the 95% CI of ∑FICmin was smaller than 0.5, significant synergy was claimed; when the 95% CI of ∑FICmax was higher than 4, significant antagonism was claimed; in all other cases, additivity was concluded. The same analysis was performed with the cutoffs 1 for ∑FICmin and 2 for ∑FICmax.
(ii) **Bliss independence response surface (BIRS) analysis.** The BI theory is described by the equation *I_i_* = *I_A_* + *I_B_* − *I_A_* × *I_B_*, where *I_i_* is the predicted percentage of inhibition of the theoretical non-interactive combination of drugs A and B, and *I_A_*, *I_B_* are the experimental percentages of inhibition of each drug acting alone, respectively. Since *I* = 1 − *E*, where *E* is the percentage of growth, by substituting into the former equation, the following equation is derived: *E_i_* = *E_A_* × *E_B_*, where *E_i_* is the predicted percentage of growth of the theoretical noninteractive combination of drugs A and B, respectively, and *E_A_* and *E_B_* are the experimental percentages of growth of each drug acting alone, respectively. The interaction is described by the difference (Δ*E*) between the predicted and measured percentages of growth with drugs at various concentrations. Because of the nature of combination testing using microtiter plates with two-fold dilution of either drug, this results in a Δ*E* for each drug combination. For each combination of the two drugs in each replicate–center experiment, the observed percent growth obtained from the experimental data was subtracted from the predicted percentage, calculated as described above. When the average difference was positive and its 95% CI among the replicates and centers did not include 0, SS synergy was claimed; when the difference was negative and its 95% CI did not include 0, SS antagonism was claimed. In any other case, BI was concluded. In order to summarize the interaction, the sum percentage of all SS synergistic (∑SYN) and antagonistic (∑ANT) interactions was calculated. Interactions with <50% of SS interactions were considered weak, those with 50% to 100% of SS interactions were considered moderate, and those with >100% of SS interactions were considered strong, as was found previously [11]. In addition, the numbers of SS synergistic and antagonistic combinations among the 49 (7 × 7) combinations tested were calculated for each strain.

**Comparisons of the FIC index with BIRS analysis.** The results of the FIC index and BIRS analysis were compared with Spearman’s correlation analysis and Fisher’s exact test. The FIC_min_s were correlated with the sum of the Bliss synergistic interaction, and the FIC_max_s were correlated with the sum of Bliss antagonistic interactions for all center replicates and strains and drug combinations. Furthermore, different FIC index and Bliss interaction sum cutoffs were used to associate the results of the two tests with Fisher’s exact test.

## 3. Results

**Minimum inhibitory concentrations (MIC) of drugs alone.** Table 1 shows the visually determined MICs of each antifungal drug against the *Candida* strains. The MICs of AB ranged from 0.5 to 4 mg/L for all strains, while for echinocandins, the MICs ranged from 0.03 to 0.5 mg/L for most strains except the two *C. parapsilosis* for which the MICs of all echinocandins ranged from 0.5 to 4 mg/L. The MIC of CAS against one *C. albicans* strain was 1–2 mg/L. The MICs of POS and VOR for the *C. albicans* and *C. glabrata* strains ranged from 0.25 to 2 mg/L, whereas the MICs for *C. parapsilosis* were lower, 0.015–0.5 mg/L. Thus, the MICs of the selected strains expanded to an extended range of drug concentrations.

**FIC index analysis.** The results of FIC index analysis are shown in Table 2 where the ranges of ∑FIC_min_ and ∑FIC_max_ of all replicates and centers are presented separately in order to capture both synergistic and antagonistic interactions, respectively, for each combination and strain. FICs obtained from most centers and strain–drug combinations except for *C. glabrata* #93 for which an FIC index was calculated by only one center for MIF+AB, CAS+MIF, POS+MIF, VOR+MIF, MIF+ANI combination because of off-scale MICs of drugs alone and in combination. For the same reason, only two centers provided FICis for *C. parapsilosis* #94 and VOR+AB, VOR+CAS, VOR+MIF, VOR+ANI combinations. The median (range) coefficients of variation for all drug combinations and strains were 13% (44–210%) for ∑FIC_min_ and 52% (14–228%) for ∑FIC_max_. The FICi differences obtained among all centers and replicates was less than two-fold (e.g., FICi ranged between 0.5 and 1, 1 and 2, etc.) for 90% of all combinations and strains with the remaining 10% showing up to four-fold differences. None of the FICis were statistically significantly lower than 0.5 or higher than 4, indicating no significant interactions. However, when the cutoffs 1 for ∑FIC_min_ and 2 for ∑FIC_max_ were used, significant pharmacodynamic interactions were found.

Based on the latter analysis, all ECH+AB combinations were found to be synergistic against all *Candida* strains except *C. glabrata* #93; no antagonism was found. For the AZO+AB combinations, synergy was found mostly with the POS+AB combination while no antagonism was found. The AZO+ECH combinations except for POS+CAS were synergistic against all *Candida* strains although with variable magnitude; significant antagonism was found for the POS+MIF combination against *C. albicans* #91. The AZO+AZO combination was additive for all strains except for a *C. parapsilosis* strain for which antagonism was also observed. ECH+ECH combinations were synergistic for all *Candida* strains except *C. glabrata* for which they were additive; no antagonism was found.

**BIRS analysis.** The results of Bliss independence response surface analysis are shown on Table 3 where the sum and the number of synergistic and antagonistic interactions are presented for each antifungal combination and *Candida* strain. The median (range) coefficient variation was 104% (49–227%) for the sum of synergistic interactions and 137% (58–245%) for the sum of antagonistic interactions.

**Figure 1:**
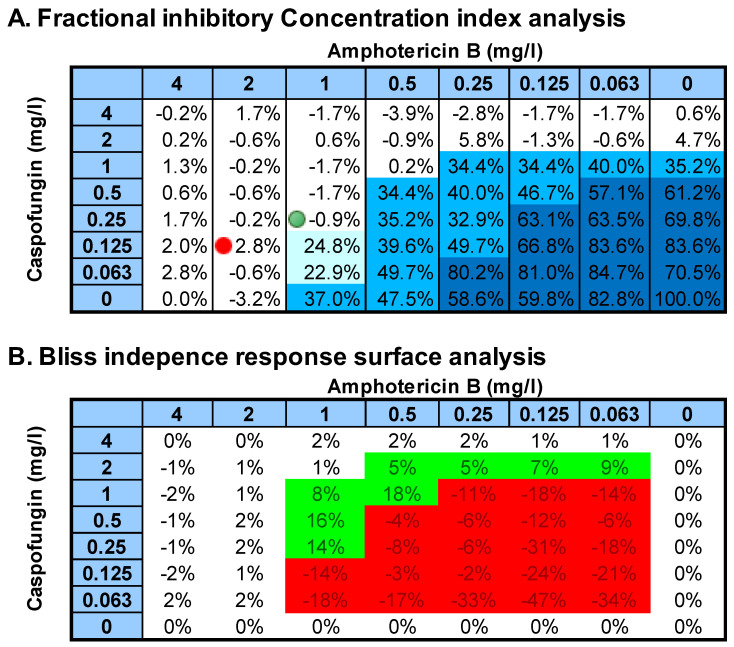
Schematic representation of Fractional inhibitory concentration (FIC) index analysis (**A**) and Bliss independence response surface analysis (**B**) of the caspofungin+amphotericin B combination against the *C. albicans* #92 strain tested in center 2. (**A**) The numbers in the cells are percentages of growth. ∑FICmin (green dot) was 0.625, and ∑FICmax (red dot) was 1.06. (**B**). The numbers in the cells are percentages of Bliss interactions. Green-colored cells represent synergistic interactions, whereas red-colored cells represent antagonistic interactions. Note that FIC index analysis captures pharmacodynamic interaction at concentrations near MIC, whereas Bliss interaction analysis captures interactions at the entire range of concentrations. Interactions at lower concentrations could be captured with FIC index using MIC-1, MIC-2, and MIC-3 corresponding to 25%, 50%, and 75% growth, shown by different shades of blue.

For the ECH+AB combinations, moderate and strong synergistic interactions were found for all *Candida* strains except *C. glabrata* (Figure 1). The most and strongest synergistic interactions were found for *C. parapsilosis* strains. Few weak antagonistic interactions were found for the CAS+AB combination. For AZO+AB combinations, weak to moderate synergistic interactions were found for all strains, while moderate and strong antagonistic interactions were found for both *C. albicans* strains. The antagonistic interactions against *C. albicans* were observed at low POS and high VOR concentrations with sub-MIC AB concentrations. AZO+AB against a *C. glabrata* strain was synergistic without antagonistic interactions. The AZO+ECH combination demonstrated strong synergistic interactions for *C. parapsilosis* strains, followed by that in a *C. albicans* strain particularly with an AZO+ANID combination, and moderate synergistic interactions were found for a *C. glabrata* strain. No antagonistic interactions were found for the AZO+ECH combination against *C. parapsilosis* and *C. glabrata*, but against *C. albicans*, antagonistic interactions were found, particularly for AZO+CAS combinations. These interactions occurred at low concentrations of ECH. The AZO+AZO combination had both weak to moderate synergistic and antagonistic interactions for *C. albicans* and *C. glabrata* strains, whereas for *C. parapsilosis*, strong antagonistic interactions were found. The latter interactions were observed near MIC resulting in an increase of AZO MICs by one dilution when combined. The ECH+ECH combinations were synergistic against *C. albicans* strains and in larger magnitude against *C. parapsilosis* strains, while weak synergistic interactions were found against *C. glabrata*.

**Figure 2:**
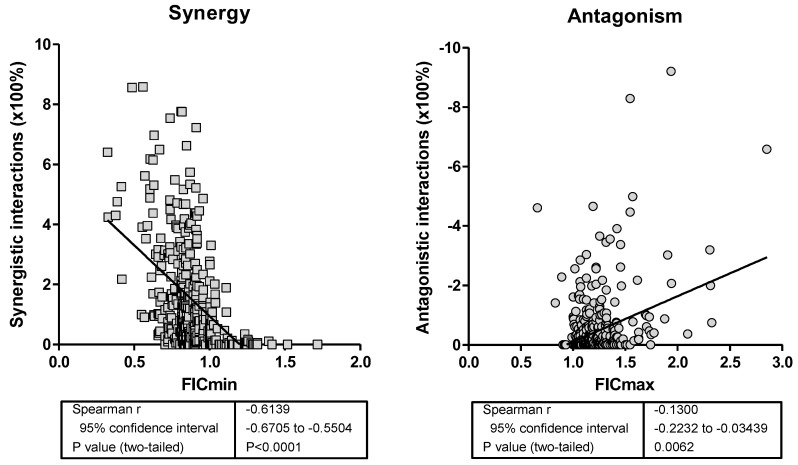
Spearman’s correlation analysis between ∑FIC_min_ and Bliss antagonistic interactions (left graph) and ∑FIC_max_ and Bliss antagonistic sum interactions (right graph).

**Comparisons of FIC index with BIRSA analysis.** The ∑FIC_min_s were strongly correlated with the sum of Bliss synergistic interactions (r_s_ = −0.61, *p* < 0.0001), and the ∑FIC_max_s were correlated with the sum of Bliss antagonistic interactions (r_s_ = −0.13, *p* < 0.0062) (Figure 2). A large number of data sets with ∑FIC_min_ between 0.5 and 1 showed statistically significantly strong (up to 800%) Bliss synergistic interactions, whereas a large number of data sets with ∑FICmax 1–4 showed statistically significantly strong (up to −1000%) Bliss antagonistic interactions. When all results were analyzed together, a strong association was found between the two analyses with the additivity cutoffs 1 and 2 for the FIC index analysis and the independence cutoffs 10% and −10% for the BIRS analysis (Chi square 646, *p* < 0.0001) (Table 4). The majority of combinations with ∑FICmin < 1 were Bliss synergistic (89.3%, 293/328) and the majority of combinations with ∑FIC_max_ ≥ 2 were Bliss antagonistic (80.6%, 25/31). For combinations with ∑FIC between 1 and 2, the majority of them were Bliss independent (60.9%, 320/525) with the remaining combinations being Bliss antagonistic (31.8%, 167/525), and some of them Bliss synergistic (7.2%, 38/525).

## 4. Discussion

Standardization of antifungal combination testing is necessary in order to reproducibly and reliably assess pharmacodynamic interactions between antifungal drugs. A multicenter study was conducted in order to determine the pharmacodynamic interactions of nine antifungal drugs in two-drug combinations against five *Candida* strains with a colorimetric broth microdilution assay [13], and the results were analyzed in the present study with two types of analyses; the most commonly used fractional inhibitory concentrations (FIC) index analysis and the alternative Bliss independence response surface (BIRS) analysis. The variation of FIC indices calculated for 12 center-replicates was mostly less than one two-fold for each strain–combination. Although the individual FIC indices ranged from 0.02 to 16, no statistically significant difference from the proposed additivity range of 0.5–4 was found, indicating no significant pharmacodynamic interactions for all of the strain–combinations tested. The median ∑FIC_min_s found in the present study were in excellent agreement (differences < 0.5) with a previous study where the FICis of the same datasets were determined indicating that the analytical bias is minimal [13]. However, BIRS analysis showed that there were significant pharmacodynamic interactions. Notably, the magnitude of BIRS interactions was strongly associated with the FIC indices. Using a narrower additivity range of 1–2 FIC index analysis, statistically significant pharmacodynamic interactions were also found and in agreement with those found with BIRS analysis.

Although several FIC index additivity ranges have been proposed in the literature, there is no consensus about which is correct [6]. The cut-offs 0.5 and 4 were suggested for defining additivity because of the two-fold drug dilution scheme and the one dilution error of single-drug susceptibility testing methods used for FIC index determination [6,16,17]. However, the actual variation of the FIC index in checkerboard microdilution methods is usually less than one two-fold dilution, and FICs seldom range from synergistic to antagonistic FICs as it was found in the present multicenter study and in previous studies [7,12]. In addition, most in vitro combination studies resulted in FIC indices within the range of 0.5–4 concluding no interaction (additivity) raise questions about the validity of this arbitrarily chosen additivity range given the absence of in vitro–in vivo correlation studies. The FIC index 0.5 is not a natural cutoff like 1 which derives from Loewe additivity theory. An additive combination of two drugs at 0.5 × MICs should result in an effect the same as that at the MIC (e.g., complete growth inhibition). If less (e.g., 0.5 × MIC of drug A and 0.25 × MIC of drug B) or more (e.g., 1 × MIC of drug A and 1 × MIC of drug B) drug is required to inhibit fungal growth completely, a synergistic and an antagonistic interaction, respectively, should take place with ∑FIC_min_ of 0.75 and ∑FIC_max_ of 2, respectively.

Overall, the ECH+AB combination was synergistic against *C. albicans* and *C. parapsilosis* but not against *C. glabrata*. This is in agreement with previous in vitro studies where MIF+AB was synergistic against *C. albicans* and *C. parapsilosis* but not against *C. glabrata* [18]. The combination CAS+AB against *C. parapsilosis* was found to be synergistic in vivo, and the FIC indices in vitro ranged between 0.5 and 1 [19]. The same combination against *C. glabrata* resulted in FIC indices of 1.03–1.09 in vitro and in additive interactions in vivo [20]. However, combination therapy with ECH+AB improved survival and reduced the fungal burden in an experimental *C. glabrata* infection, emphasizing that an additive in vitro combination can result in an improved outcome in vivo [21,22].

The AZO+AB combination and in particular the POS+AB combination were synergistic against most of the strains. Antagonistic interactions were found with both *C. albicans* strains with BIRS analysis but not with the FIC index analysis. BIRS analysis assesses interactions at the entire range of drug concentrations tested, whereas the FIC index analysis assess pharmacodynamic interactions only at the MIC level as it is illustrated in Figure 1. The antagonistic interactions detected with BIRS analysis were found at concentrations lower than MIC and could be detected with the FIC index analysis if another MIC endpoint was used, e.g., MIC-2 corresponding to 50% of growth inhibition as was found previously [12].

The AZO+ECH combination was synergistic against all *Candida* strains. Although the magnitude of synergistic interactions was the same for all strains with the FIC index analysis, the BIRS analysis showed much more synergistic interactions against *C. parapsilosis* followed by *C. albicans*, and the least synergistic interactions were found against *C. glabrata*. BIRS analysis also showed strong antagonistic interactions for the AZO+ECH combination against *C. albicans* strains. Because of the noncontinuous nature of the FIC indices (e.g., there are no FICs between 1.5 and 2 and between 0.75 and 1), they tend to cluster around certain values and therefore their discriminatory power to distinguish between similarly synergistic and antagonistic interactions is small. By contrast, BIRS analysis can detect even small differences of pharmacodynamic interactions because the entire response-surface was analyzed. Previous in vitro combination studies of AZO+ECH reported FICs between 0.5 and 1 as in the present study, concluding indifference, or more correctly, additivity based on the additivity range of 0.5–4 [23,24].

The intraclass combination AZO+AZO was additive for all strains, and some antagonistic interactions were found for one *C. parapsilosis.* The intraclass ECH+ECH combination was synergistic for all *Candida* strains, with *C. parapsilosis* showing the strongest synergistic interactions. Different susceptibilities to echinocandins have been reported for *C. parapsilosis*, indicating the site of action may be different [25]. The different site of actions may explain the synergistic interactions found in the present study with ECH–ECH combinations.

In conclusion, the present study demonstrated that standardization of combination antifungal susceptibility testing is feasible. An important factor towards this direction is replication which would help assess more reproducibly pharmacodynamic interactions. The FIC index cutoffs of 0.5 to 4 may be too wide to capture significant pharmacodynamic interactions; an additivity range of 1–2 with replication may be more useful to assess results of combination testing. BIRS analysis assesses interactions at the entire range of drug concentrations tested and can detect even small differences of pharmacodynamic interactions at sub-MIC concentrations which are important since in vivo drug levels fluctuate over time. The interclass combinations ECH+AB and ECH+AZO were synergistic particularly for *C. parapsilosis;* ECH+AZO showed some antagonistic interactions against *C. albicans*. The AZO+AB combination was synergistic for all strains, while some antagonistic interactions were also found against *C. albicans*. The intraclass combination AZO+AZO was additive, whereas the ECH+ECH combination was synergistic.

## Figures and Tables

**Table 1:** The minimal inhibitory concentrations of six antifungal drugs against the five test *Candida* isolates determined with the Sensititre Yeast colorimetric broth microdilution test.

Antifungal Drug	*C. albicans* #91 (20533.043)	*C. albicans* #92 (20464.007)	*C. glabrata* #93 (20205.075)	*C. parapsilosis* #94 (20580.070)	*C. parapsilosis* QC ATCC 22019
Amphotericin B	1 (0.5–2)	1 (0.5–2)	1 (0.5–2)	1 (0.5–4)	1 (0.5–2)
Anidulafungin	0.06 (0.03–0.06)	0.25 (0.12–0.5)	0.06 (0.03–0.06)	2 (2–4)	2 (0.5–2)
Caspofungin	0.12 (0.06–1)	1 (1–2)	0.06 (0.06–0.12)	1 (0.5–1)	1 (0.5–2)
Micafungin	0.06 (0.03–0.12)	0.12 (0.12–0.5)	0.06 (0.06–0.5)	2 (1–2)	2 (1–4)
Posaconazole	1 (0.5–2)	0.5 (0.25–1)	2	0.12 (0.06–0.25)	0.25 (0.12–0.5)
Voriconazole	2 (1–2)	2	2	0.015	0.03 (0.03–0.12)

**Table 2:** Results of the Loewe additivity-based FICi index analysis of two-drug combinations between echinocandins, azoles, and amphotericin B obtained from the 6 centers for each of the 5 *Candida* isolates tested.

Combinations ^a^	Drugs ^b^	*C. albicans* #91	*C. albicans* #92	*C. glabrata* #93	*C. parapsilosis* #94	*C. parapsilosis* QC
**-------------------------------------------------------LOEWE SYNERGISTIC INTERACTIONS (∑FIC_min_)------------------------------**						
**Interclass combinations**						
ECH+AB	ANI+AB	0.44 (0.09–0.63) ^c^	0.5 (0.31–0.75) ^c^	0.63 (0.5–1.13)	0.52 (0.04–1) ^c^	0.75 (0.16–1) ^c^
	CAS+AB	0.56 (0.19–1.06) ^c^	0.63 (0.53–1.06) ^c^	1 (0.5–1.06)	0.75 (0.13–1.25) ^c^	0.66 (0.19–1.13) ^c^
	MIF+AB	0.53 (0.31–0.56) ^c^	0.5 (0.16–0.75) ^c^	0.63 (0.63–0.63)	0.56 (0.16–1.13) ^c^	0.55 (0.19–1.06) ^c^
AZO+AB	POS+AB	0.64 (0.14–1.03) ^c^	0.34 (0.19–1.03) ^c^	0.52 (0.04–1) ^c^	0.53 (0.25–1.13) ^c^	1 (0.31–1.25)
	VOR+AB	0.78 (0.13–2.01)	1.01 (0.51–1.06)	1.01 (0.31–1.02)	0.28 (0.19–0.56) ^c^	0.81 (0.38–1.5)
AZO+ECH	VOR+CAS	0.59 (0.31–1) ^c^	0.53 (0.51–1.01) ^c^	0.51 (0.26–1) ^c^	0.13 (0.05–0.56) ^c^	0.55 (0.28–1.06) ^c^
	POS+CAS	1 (0.28–2)	0.28 (0.09–1.5) ^c^	0.63 (0.31–1.03)	0.52 (0.09–1.5) ^c^	1 (0.07–2)
	POS+MIF	0.53 (0.51–1.02) ^c^	0.51 (0.25–1.03) ^c^	0.54 (0.51–0.56) ^c^	0.55 (0.05–0.75) ^c^	0.59 (0.38–1.06) ^c^
	VOR+MIF	0.52 (0.51–0.75) ^c^	0.51 (0.26–1.01) ^c^	0.51 (0.51–0.52) ^c^	0.06 (0.04–0.53) ^c^	0.75 (0.25–1.06) ^c^
	POS+ANI	0.51 (0.03–1.06) ^c^	0.44 (0.09–1.03) ^c^	0.51 (0.13–2.01) ^c^	0.41 (0.02–0.56) ^c^	0.53 (0.28–1) ^c^
	VOR+ANI	0.52 (0.05–1.02) ^c^	0.51 (0.26–1.01) ^c^	0.53 (0.14–2.01) ^c^	0.05 (0.02–0.52) ^c^	1.02 (0.52–2.06)
**Intraclass combinations**						
AZO+AZO	POS+VOR	1 (0.08–2.01)	1 (0.13–1.5)	ND	0.63 (0.38–3)	1.25 (0.63–2.06)
ECH+ECH	CAS+MIF	0.56 (0.5–1) ^c^	0.5 (0.28–0.63) ^c^	1	0.75 (0.25–1) ^c^	0.59 (0.14–2) ^c^
	MIF+ANI	0.75 (0.52–1.5)	0.5 (0.38–0.56) ^c^	0.88 (0.75–1)	0.53 (0.38–1) ^c^	0.69 (0.14–1.25) ^c^
	ANI+CAS	0.56 (0.16–1) ^c^	0.38 (0.25–0.63) ^c^	1 (0.38–1)	0.63 (0.06–1.03) ^c^	0.75 (0.5–1.25) ^c^
**------------------------------------------------------LOEWE ANTAGONISTIC INTERACTIONS (∑FIC_max_)---------------------------**						
**Interclass combinations**						
ECH+AB	ANI+AB	1.25 (1.02–1.5)	1.25 (1.06–2.06)	1.5 (1.25–3)	1.25 (1.06–2.03)	1.5 (1.02–4.25)
	CAS+AB	1.25 (1.01–4.25)	1.25 (1.13–4.25)	1.5 (1.5–5)	2.13 (1.06–4.25)	1.5 (1.03–5)
	MIF+AB	1.5 (1.25–1.5)	1.25 (1.03–4.13)	1.81 (1.5–2.13)	1.5 (1.13–2.5)	1.25 (1.03–4.25)
AZO+AB	POS+AB	1.38 (0.56–2.5)	1.19 (1.01–4.06)	1.25 (1.01–2.5)	1.25 (1.01–4.02)	2.25 (1.13–4.13)
	VOR+AB	1.5 (0.63–2.5)	2.13 (1.5–4.02)	1.5 (1.01–2.5)	1.5 (1.01–2.5)	2.5 (1.25–4.03)
AZO+ECH	VOR+CAS	1.5 (1.01–2.5)	1.5 (1.01–4.06)	1.5 (1.01–2.1)	1.01 (1.01–1.5)	2.13 (1.5–4.5)
	POS+CAS	1.5 (1.01–4.13)	1.03 (1.01–3)	1.13 (1.01–2.5)	1.5 (1.01–4.5)	2 (1.06–4.5)
	POS+MIF	3 (1.5–8.5) ^d^	1.13 (1.01–2.5)	1.77 (1.5–2.03)	1.25 (1.06–2.5)	1.78 (1.06–2.5)
	VOR+MIF	1.5 (1.01–4.5)	1.5 (1.01–2.25)	1.01 (1.01–1.02)	1.27 (1.01–2.01)	2 (1.13–4)
	POS+ANI	1.5 (1.01–4.25)	1.05 (1.01–4.02)	1.01 (1.01–4.06)	1.19 (0.53–2.13)	1.5 (1.5–4.5)
	VOR+ANI	2.25 (1.01–4.5)	1.02 (1.01–2.5)	1.13 (1.01–4.02)	1.03 (0.51–1.5)	2.19 (1.5–5)
**Intraclass combinations**						
AZO+AZO	POS+VOR	1.5 (1–2.5)	1 (0.63–2.25)	ND	1.88 (1.13–4.13)	2.75 (1.5–9) ^d^
ECH+ECH	CAS+MIF	1.5 (1.25–1.5)	1.13 (1.13–4.25)	1.5 (1.5–1.5)	1.5 (1.03–4.03)	1.5 (0.63–4.5)
	MIF+ANI	1.5 (1.25–2.5)	1.25 (1.13–4.25)	1.5 (1.5–1.5)	1.25 (1.01–1.5)	1.5 (1.25–4.5)
	ANI+CAS	1.5 (1.25–2.5)	1.13 (1.03–2.25)	1.5 (1.25–4.5)	1.19 (1.01–3)	1.5 (1.13–4.25)

^a.^ ECH, echinocandins; AZO, azole; ^b.^ ANI, Anidulafungin; AB, amphotericin B; CAS, caspofungin; MIF, micafungin; POS, posaconazole; VOR, voriconazole. ^c^ ∑FICmin significantly smaller than 1 (*p* < 0.05). ^d^ ∑FICmax significantly higher than 2 (*p* < 0.05).

**Table 3:** Results of Bliss independence-based response surface analysis of two-drug combinations between echinocandins, azoles, and amphotericin B obtained from the 6 centers for each of the 5 *Candida* isolates tested.

Combination ^c^	Drugs ^b^	*C. albicans* #91		*C. albicans* #92		*C. glabrata* #93		*C. parapsilosis* #94		*C. parapsilosis* QC	
		Sum	(N)	Sum	(N)	Sum	(N)	Sum	(N)	Sum	(N)
**------------------------------------------------------------------BLISS SYNERGISTIC INTERACTIONS-----------------------------------**											
**Interclass combinations**											
ECH+AB	ANI+AB	138%	(23)	74%	(30)	10%	(19)	364%	(35)	169%	(29)
	CAS+AB	64%	(16)	34%	(20)	21%	(26)	116%	(34)	60%	(27)
	MIF+AB	74%	(17)	88%	(31)	14%	(24)	221%	(30)	281%	(32)
AZO+AB	POS+AB	143%	(20)	35%	(17)	131%	(38)	44%	(24)	35%	(26)
	VOR+AB	93%	(14)	13%	(16)	42%	(15)	70%	(33)	14%	(11)
AZO+ECH	VOR+CAS	110%	(13)	34%	(14)	44%	(22)	398%	(47)	116%	(25)
	POS+CAS	23%	(9)	60%	(18)	35%	(29)	321%	(37)	83%	(35)
	POS+MIF	22%	(6)	158%	(40)	56%	(36)	269%	(30)	175%	(21)
	VOR+MIF	40%	(11)	249%	(38)	57%	(33)	490%	(37)	70%	(28)
	POS+ANI	171%	(24)	136%	(30)	25%	(12)	452%	(40)	135%	(24)
	VOR+ANI	135%	(28)	234%	(42)	51%	(23)	578%	(49)	10%	(19)
**Intraclass combinations**											
AZO+AZO	POS+VOR	84%	(7)	117%	(21)	16%	(4)	0%	(2)	4%	(4)
ECH+ECH	CAS+MIF	27%	(8)	150%	(28)	21%	(31)	223%	(36)	161%	(19)
	MIF+ANI	0%	(0)	138%	(28)	6%	(23)	397%	(33)	260%	(19)
	ANI+CAS	38%	(10)	112%	(27)	15%	(25)	177%	(24)	70%	(14)
**----------------------------------------------------------------------BLISS ANTAGONISTIC INTERACTIONS---------------------------**											
ECH+AB	ANI+AB	−2%	(1)	0%	(0)	0%	(0)	−2%	(2)	−7%	(1)
	CAS+AB	−40%	(6)	−33%	(2)	−23%	(3)	0%	(0)	−30%	(2)
	MIF+AB	−2%	(2)	0%	(0)	0%	(0)	0%	(0)	−2%	(1)
AZO+AB	POS+AB	−100%	(9)	−68%	(4)	0%	(0)	−6%	(1)	−14%	(1)
	VOR+AB	−71%	(8)	−172%	(12)	0%	(0)	0%	(0)	−2%	(2)
AZO+ECH	VOR+CAS	−234%	(17)	−161%	(10)	0%	(0)	0%	(0)	0%	(0)
	POS+CAS	−333%	(24)	−99%	(5)	0%	(0)	0%	(0)	0%	(0)
	POS+MIF	−66%	(13)	0%	(0)	−1%	(1)	0%	(0)	−5%	(3)
	VOR+MIF	−51%	(9)	0%	(0)	0%	(0)	0%	(0)	−4%	(3)
	POS+ANI	−12%	(4)	0%	(0)	0%	(0)	0%	(0)	0%	(0)
	VOR+ANI	−47%	(7)	0%	(0)	0%	(0)	0%	(0)	−2%	(1)
AZO+AZO	POS+VOR	−15%	(1)	−78%	(5)	−35%	(3)	0%	(0)	−293%	(11)
ECH+ECH	CAS+MIF	−5%	(3)	0%	(0)	0%	(0)	0%	(0)	0%	(0)
	MIF+ANI	−9%	(5)	0%	(0)	−2%	(1)	0%	(0)	0%	(0)
	ANI+CAS	−4%	(3)	0%	(0)	−2%	(1)	0%	(0)	−93%	(5)

^c^ ECH, echinocandins; AZO, azole; ^b^ ANI, Anidulafungin; AB, amphotericin B; CAS, caspofungin; MIF, micafungin; POS, posaconazole; VOR, voriconazole.

**Table 4:** Chi square analysis of the FIC index and BIRS analysis.

Loewe Additivity Interactions	Bliss Independence Interactions		
	SYN (≥10%)	IND (<10%, >−10%)	ANT (≤−10%)
SYN (FIC < 1)	293	31	4
ADD (FIC 1–2)	38	320	167
ANT (FIC ≥ 2)	0	6	25

Chi-square, df = 646.1, 4, *p* < 0.0001.

## Data Availability

Data available on request.
